# Contact Force Measurement in an Operational Thrust Bearing using PVDF Film at the Blade and Pad Passing Frequencies

**DOI:** 10.3390/s18113956

**Published:** 2018-11-15

**Authors:** Andrew Youssef, David Matthews, Andrew Guzzomi, Jie Pan

**Affiliations:** School of Engineering, The University of Western Australia, 35 Stirling Hwy, Crawley, WA 6009, Australia; andrew.youssef@research.uwa.edu.au (A.Y.); david.matthews@uwa.edu.au (D.M.); andrew.guzzomi@uwa.edu.au (A.G.)

**Keywords:** PVDF, monitoring, thrust bearing, force transducer

## Abstract

A major contributor to longitudinal vibration in marine propulsion systems is propeller induced excitation. This constitutes a key source of underwater acoustical radiation through excitation of the hull. Understanding this hydrodynamic force at the interface of the thrust bearing is important in order to develop an accurate vibrational model of the propulsion system and in determining potential control mechanisms. In order to investigate the thrust force during operation of a propulsion system, Polyvinylidene Fluoride (PVDF) was embedded into the stationery collar inside a custom thrust bearing in a scaled model of a typical propulsion system. The number of blades of the propeller and its rotational speed were altered to obtain an understanding of the characteristic vibrations of the shaft propulsion system. The rig comprised of the propeller, shaft, journal bearings and a thrust bearing. A two and three blade propeller and a four, five and six pad bearing were tested. A strain gauge and accelerometer were used to infer the propeller force and enable comparison with the PVDF signals. As a result of the asymmetrical flow around the propeller, the blade passing frequencies (BPF) are clearly observed. This frequency contribution was present at all speeds tested. The PVDF signal also showed significant pad passing frequency (PPF) and BPF and modulation of both.

## 1. Introduction

The propulsion system of marine vessels is a major vibration source as it couples the vessel to the motor. Flow noise and cavitation at the propeller produce a broadband spectrum of noise. In submerged vessels operating in deep water, cavitation is not prevalent due to the increased water pressure. Propeller noise is generated when a propeller is rotating through a non-uniform flow due to asymmetries of the hull and other protrusions in the vicinity of the propeller’s flow field which tend to produce vibrations at frequencies below 100 Hz [1]. The turbulent flow means different parts of the propeller are subjected to varying forces and pressure distributions from the water. This in turn means the blades are producing varying magnitudes of thrust as the propeller rotates, transmitting vibrations into the shafting system and surrounding fluid. A significant contribution to these vibrations is produced at a frequency known as the blade passing frequency (BPF) which is equal to the shaft rotational frequency multiplied by the number of blades of the propeller and an integer representing the order of the BPF. The fluctuating forces caused by the propeller occur in the radial, tangential and axial directions with the radial and tangential components being of similar magnitude maybe coupled through journal bearings [1]. Nevertheless, the axial variations in thrust transmitted along the shaft generate hull vibrations through the hydrodynamic stiffness of the thrust bearing. The vessel then produces noise due to excitation of the hull arising from the shafting system.

The thrust bearing stiffness has a major influence on the vibration transmission into the hull. However, producing accurate models of the bearing can be difficult. Whilst some works [1,2,3] assume a simple linear stiffness for the thrust bearing, it has been shown [4,5,6] that the bearing exhibits nonlinear characteristics under high load due to large perturbations of the oil film thickness found in the bearing. It is clear that accurate models of the thrust bearing are vital in order to predict the vibrational response of the propulsion system.

In our previous paper, Reference [7], an in depth review of the use of PVDF to measure dynamic forces in static structures was discussed [8,9,10,11,12,13,14,15]. We also reported on the feasibility of using PVDF inside a model thrust bearing to measure the pressure fluctuations and contact forces. Although that study found that the sensors could measure the pressure generated by the sliding pads, the PVDF was embedded inside a simplified thrust bearing that did not carry a realistic load such as one generated by a propeller. Here, we extend the work in Reference [7] by embedding the PVDF film onto the collar inside an operational thrust bearing operating under different conditions. It is acknowledged that there is a wealth of information regarding bearing analysis as well as rotor dynamics and those fields continue to receive much research attention. However, here we focus on the potential of PVDF sensors.

## 2. Materials and Methods

**Figure 1:**
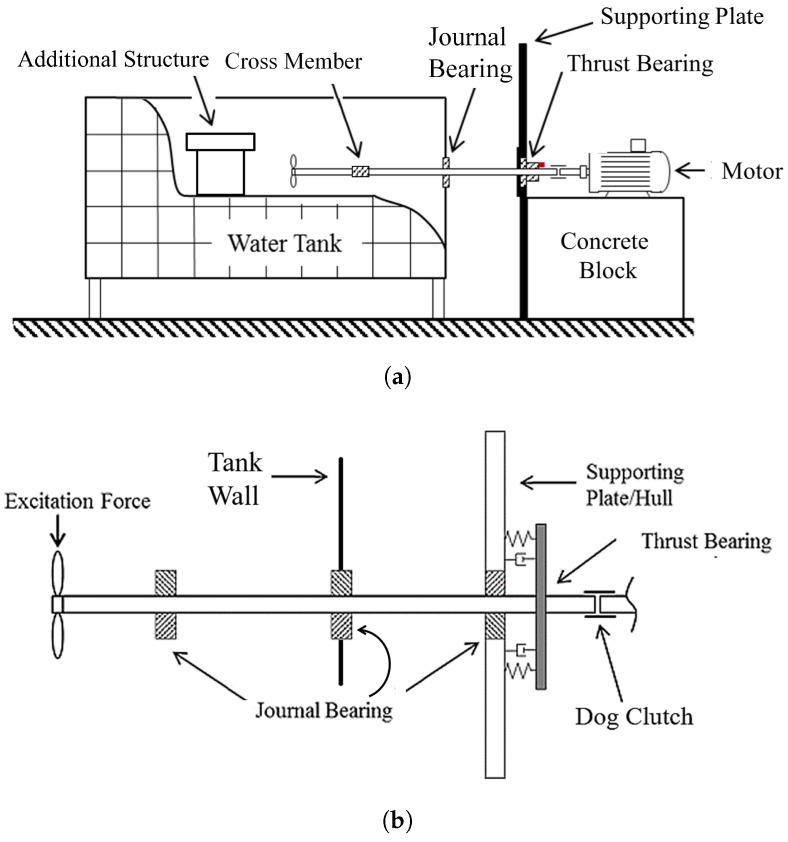
Schematic of the experimental rig (**a**), enlarged view of propulsion system (**b**).

**Figure 2:**
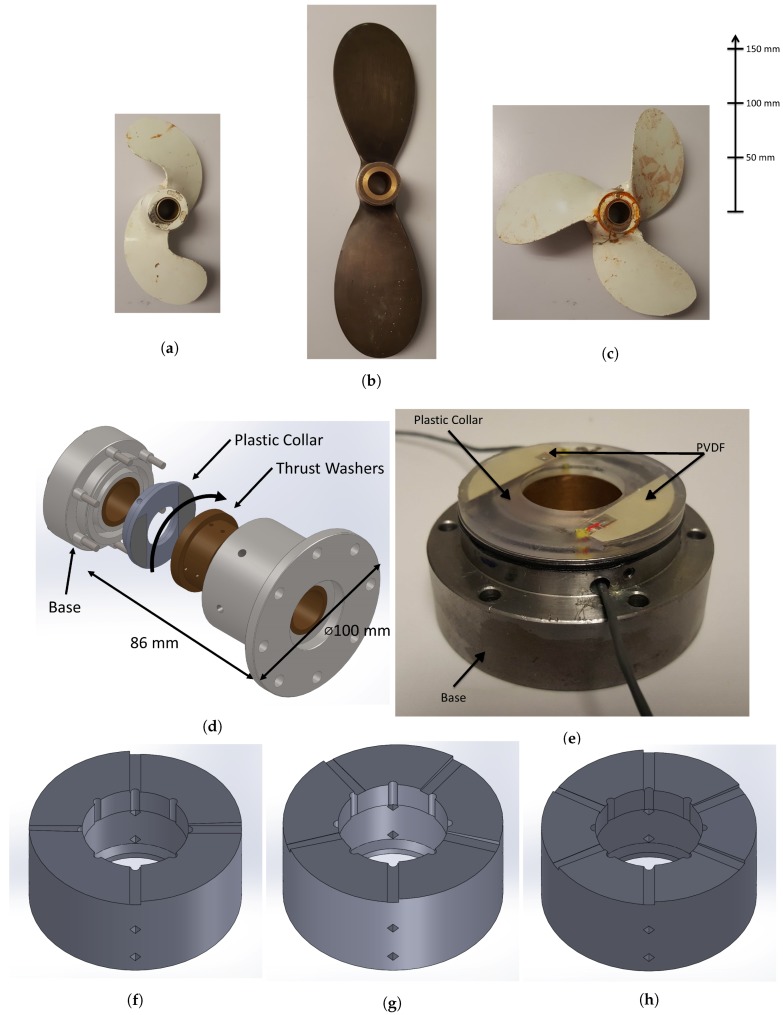
The two-blade (**a**), brass two-blade (**b**) and three-blade (**c**) propellers used in the experiment. (**d**) Assembly drawing of the thrust bearing. (**e**) The thrust bearing washer with embedded PVDF film. The four (**f**), five (**g**) and six (**h**) pad bearings used.

The rig shown in Figure 1a was used to investigate the pressure fluctuations inside the thrust bearing with changes in load and speed. The experimental set-up was designed to model a typical propulsion system as depicted in Figure 1b. The system consists of a propeller, shaft, journal bearings, and a thrust bearing attached to a supporting plate structure. The propeller provides axial thrust to the shaft which is transmitted to the supporting plate via the thrust bearing. The dog clutch coupling which joins the downstream shaft to the motor is rigid in torsion but flexible in bending and allows the downstream shaft to float so that the applied load from the propeller is transmitted to the thrust bearing. This arrangement also helps isolate unwanted vibration from the six pole AC electric motor. The supporting plate is fixed to the concrete block which also supports the motor as shown in Figure 1a. A 10 mm thick, 630 mm wide steel plate is fixed to the supporting structure to take the thrust load generated at the propeller. Two two-blade and a three-blade propeller as shown in Figure 2a–c, respectively, were separately coupled to the propulsion system and the system immersed in the water tank (0.99 × 0.59 × 0.58 m) to observe different blade passing forces. One of the two-blade propellers and the three-blade propeller had equal diameter of 0.22 m and the second-two blade propeller had a larger diameter of 0.3 m and was of an alternative style and constructed from brass. It should be noted that the shaft was supported within the water tank by a cross member impeding the inflow to the propeller resulting in a non uniform wake. In addition to this, as this is a repurposed tank, a large structure was in the water tank, downstream of the propeller further impeding flow. Both structures had the potential to assist with the generation of BPF excitation.

A total of three journal bearings are included in the rig. They have clearances of 280, 190 and 180 μm and slenderness ratios of 1, 0.42 and 0.42, respectively. These bearings are selected as they are typically found in marine propulsion systems. The parameters of the thrust bearing such as the minimum and maximum oil film thickness are load dependant and are difficult to speculate.

In order to measure the contact force inside the thrust bearing, a simple collar was constructed out of Acrylonitrile Butadiene Styrene (ABS) using a 3D printer as shown in Figure 2d. Recesses were formed in the collar to insert two PVDF films as shown. This was the simplest arrangement as the collar was stationery and its flat surface made it straightforward to attach the PVDF films. Opposite the plastic collar, a washer was also constructed out of ABS with four, five and six pads angled at two degrees to the surface. Each washer would be coupled to the rotating shaft. As the 3D printer layer height was 0.1 mm, the surface profile comprised of 11 individual steps, these are shown in Figure 2f–h, respectively. It was presumed that the forces, temperatures and operating times would be sufficiently small, so that the use of plastic material inside the thrust bearing would not be a problem.

The same technique as described in Reference [7] was used for this experiment and briefly explained here. The two PVDF sensors were cut from a sheet of 110 μm thick film purchased from Measurement Specialties [16]. Contacts were made onto the electrodes using conductive copper tape and the films were glued inside the two recesses in the plastic collar using West Systems 105 epoxy resin as shown in Figure 2e.

Coaxial cables from the sensors were fed through holes in the bearing housing. The entire bearing was then installed into the propulsion system assembly shown in Figure 2d. The outputs from the PVDF films were conditioned using a Brüel & Kjaer Type 2635 charge amplifier. The embedded PVDF films were calibrated using an impact hammer method discussed in our previous work [7]. Similar results were found for the two sensitivities of 6.8 mV/N and 7.1 mV/N. In the discussion that follows, only the 6.8 mV/N PVDF film is discussed as both sensors produced similar trends.

**Figure 3:**
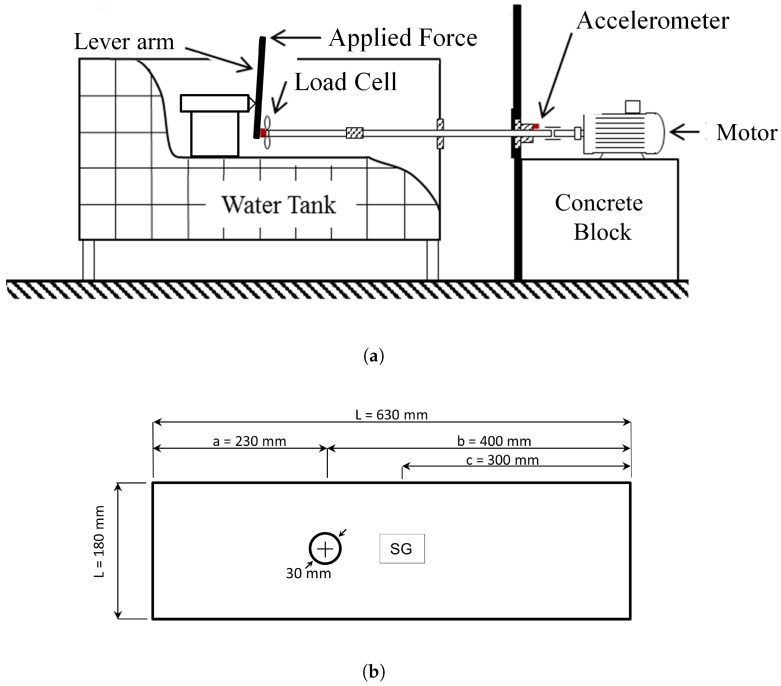
Schematic of: (**a**) the method of applying the force down the length of the shaft; (**b**) the supporting beam of the rig.

To gain further insight into the behaviour of the structure, a PCB 352C67 accelerometer was attached to the thrust bearing in the axial direction as shown in Figure 3a. A Brüel & Kjaer LAN-XI data acquisition system (Type 3050-B-060) was used in conjunction with the Brüel & Kjaer Pulse Time Data Recorder program to analyse and collect data from the PVDF and accelerometer in the time domain. The data was recorded at a sampling rate of 16,384 Hz such that any higher frequency noise from the sensors would be captured whilst keeping file sizes manageable.

A HBM strain gauge (Type 1.5/120 XY31-4L-3M) in a half-bridge configuration was placed on the 10 mm thick steel plate used to support the thrust bearing, Figure 3b, to measure the dynamic strain due to the thrust force from the propeller. The strain gauge was oriented in the shaft’s axial direction, in the primary direction of deflection, at the centreline of the shaft. The strain gauge measured both the steady thrust force as well as the fluctuating forces resulting from the non-uniform flow. The data was recorded through a HBM QuantumX (Model MX1615B) data acquisition system in conjunction with the HBM catman-easy software at the same 16,384 sampling rate. The method to estimate the propeller force from the strain gauge is discussed in the Appendix A.

First, an impact hammer test (PCB Model Number: 086C03) was performed to characterise the response of the supporting plate. This was achieved by placing an accelerometer onto the supporting plate and striking the plate measuring the frequency response function using the same previously discussed data acquisition system. This was then followed by testing five configurations of the rig. The first with the electric motor turned off to measure the noise floor of the PVDF. The second configuration corresponded to the shaft coupled to the motor without the propeller and enabled the noise transmission of the shaft spinning in the bearing to the PVDF to be observed. The last three configurations where for the three propellers shown in Figure 2a–c coupled to the shaft. Each configuration was tested at speeds from 0 RPM to 600 RPM at 20 RPM increments. At each of these shaft speeds, 10 s of data was recorded. The 10 s records were stitched together resulting in a 310 s file for each configuration, allowing a spectrogram to be produced.

## 3. Results

**Figure 4:**
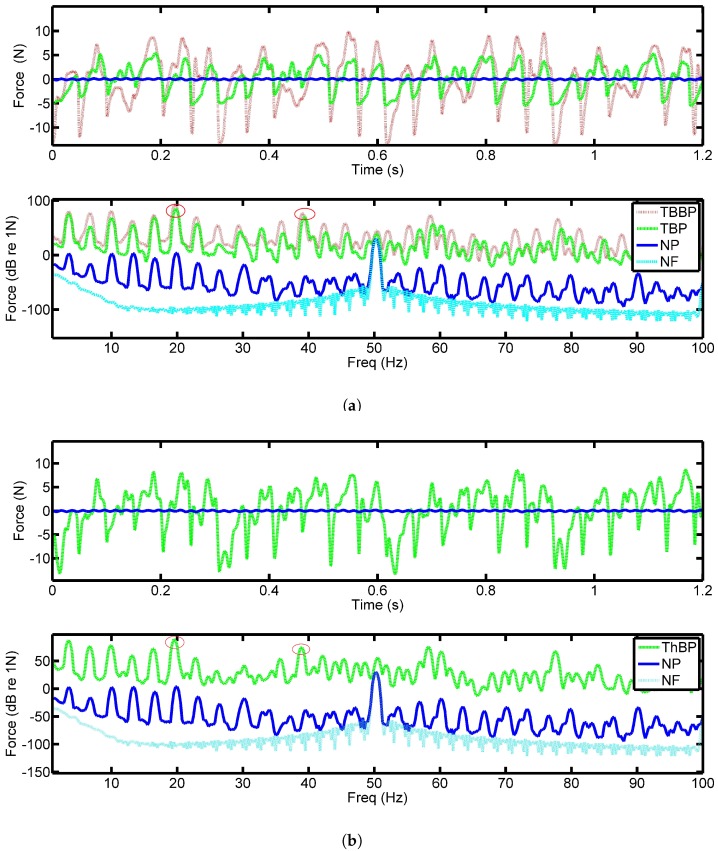
Captured force from the PVDF with the: (**a**) two-blade propeller, (**b**) three-blade propeller rotating at 200 RPM with a six-pad bearing, time (top) and frequency (bottom) data. Where: TBBP–Two-blade brass propeller, TBP–Two-blade propeller, ThBP - Three-blade propeller, NP–No propeller, NF–Noise floor.

Figure 4a shows the force output from the PVDF film when both two-blade propellers were spun at 200 RPM or 3.33 Hz with the six pad bearing. The top plot of Figure 4a shows the time series data for four rotations of the shaft. With the abscissa equal to 1.2 s, the plot shows four complete revolutions. The blue curve is the output force with no propeller on the shaft. As expected, this results in a significantly smaller output force than the two cases with the propellers attached. This confirms that the complex structure observed when the propeller is attached (which is in the red and green data sets) is due to its induced effects. It should also be noted that the brass propeller (red line) produces a significantly larger force than the smaller swept two-blade version, see Figure 2a,b.

The lower plot in Figure 4a shows the spectrum of the time domain data. The cyan plot shows the electrical noise floor of the system in the stationary state and clearly shows the presence of 50 Hz noise which corresponds to the electricity supply frequency. At all other frequencies the noise floor is well below the measured signals. The blue curve shows the plain shaft results, where the shaft rate can clearly be seen with the prominent 50 Hz peak. It can be seen that the introduction of the propeller has resulted in an increase of approximately 50 dB over the entire frequency range. It should be noted that the 6th harmonic indicated in Figure 4a is the pad passing frequency (PPF) and is a few dB larger than the rest of the data.

Shown in Figure 4b is the data captured when the three-blade propeller is used. Similar trends are observed in the two-blade propeller results. However, it is interesting to note that there appears to be little difference between all the signatures from the PVDF, suggesting that the response is dominated by the pads of the bearing. This may be due to the fact that the fluctuations of the propeller is supported by the thrust pads. The PVDF senses the impulses of the thrust pads with the superimposed propeller force making it difficult to observe. It should be noted that the response of the PVDF is independent of the application location of the force meaning a concentrated load will have the same response as a distributed load across the sensor of the same magnitude [7]. Since the purpose of the sensors was to examine the contact equivalent force inside the bearing, this result appears to be reasonable.

**Figure 5:**
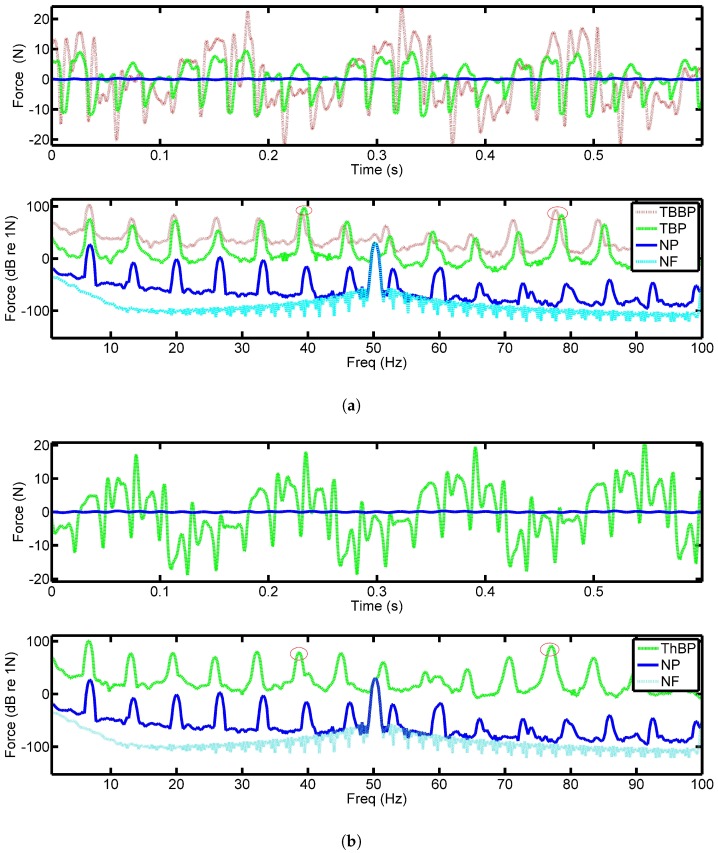
Captured force from the PVDF with the (**a**) two-blade propeller, (**b**) three-blade propeller rotating at 400 RPM with a six-pad bearing, time (top) and frequency (bottom) data. Where: TBBP–Two-blade brass propeller, TBP–Two-blade propeller, ThBP–Three-blade propeller, NP–No propeller, NF–Noise floor.

Plots in Figure 5 show similar results for a rotational speed of 400 RPM or 6.67 Hz. It can be seen that the obtained waveforms are better defined than at the lower speed of 200 RPM. This is most likely due to the formation of a higher pressure oil film under the tilted pads in the bearing and the larger thrust generated by the propeller. At lower speeds, the oil film is likely only partly formed and the pads are only partially lifted off the collar. The increase in speed clearly shows the shaft rotation in the time domain (Figure 4a) as can be seen by the amplitude modulation of the four cycles. This is especially evident in the three-blade propeller data where the larger forces produce an increase in shaft noise being transmitted into the bearing. The repeating signatures contain six peaks which are the impulses caused by the oil pressure generated underneath the pads, i.e., PPF.

The frequency domain data shows similar trends as the 200 RPM data, where the shaft rate has increased from 3.33 to 6.67 Hz. At the 400 RPM speed, the peaks in the data appear to diverge from the shaft rate’s harmonics, this is also the case for the 200 RPM data. This is due to a lag associated with the set speed of the AC motor used and the actual speed of the shaft. In addition to this, it can be seen that the change between the peaks and the shaft rate harmonics are greater when a propeller is attached compared to just the shaft alone. The additional torque introduced by the propeller results in a speed dependent lag between the nominal speed and true speed and increases with higher load.

**Figure 6:**
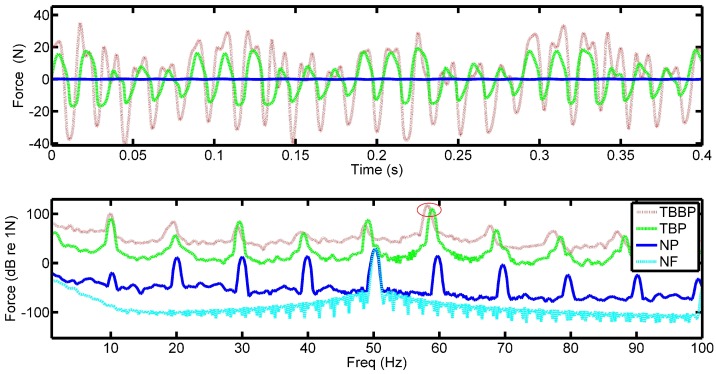
Captured force from the Polyvinylidene Fluoride (PVDF) with the two-blade propeller rotating at 600 RPM with a six pad bearing, time (top) and frequency (bottom) data. Where: TBBP–Two-blade brass propeller, TBP–Two-blade propeller, NP–No propeller, NF–Noise floor.

Figure 6 shows the result at 600 RPM or 10 Hz, the highest speed selected for the experiment. The time domain data sees an improvement in the waveform shape produced for the two-blade propellers. From the short bearing approximation [17] and the speed coupled with a small propeller, we can be confident that the oil film is well developed. This is believed to be a good representation of the pressurised oil film sliding over the PVDF film, as the developed waveform is similar to that of earlier investigations with PVDF inside a model thrust bearing discussed in our previous work [7]. The three-blade results are not included because it was found that the large loads produced by the propeller had damaged the films. This was due to irregularities in the surfaces and the small clearances inside the bearing.

**Figure 7:**
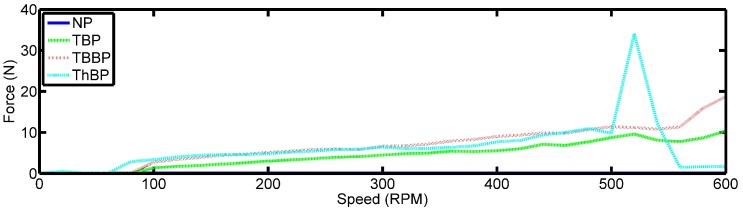
The RMS values of the time signatures captured for all speeds for each propeller configuration.

The Root Mean Square (RMS) of the time domain signatures are shown in Figure 7. There is a steady increase of the observed forces with an increase in speed. The three-blade propeller and the larger brass two-blade propeller produce the larger loads at the same speeds compared to the smaller two-blade propeller. However, there are two features of interest. Firstly, the PVDF does not sense a force at low speeds less than 100 RPM. This is believed to be caused by the small forces produced by the propeller to engage the thrust bearing at these speeds, meaning the pressure formed underneath the tilted pad is insufficient to observe a noticeable force. This hypothesis is supported by the fact that the three-blade propeller begins to sense the pressure at a slower speed of 80 RPM when compared to the two-blade propellers, as the pressure formed is a function of applied load. The second feature of interest is the large peak seen at 520 RPM for the three-blade propeller. This occurred when the epoxy in the PVDF failed resulting in delamination of the PVDF sensor as mentioned above.

**Figure 8:**
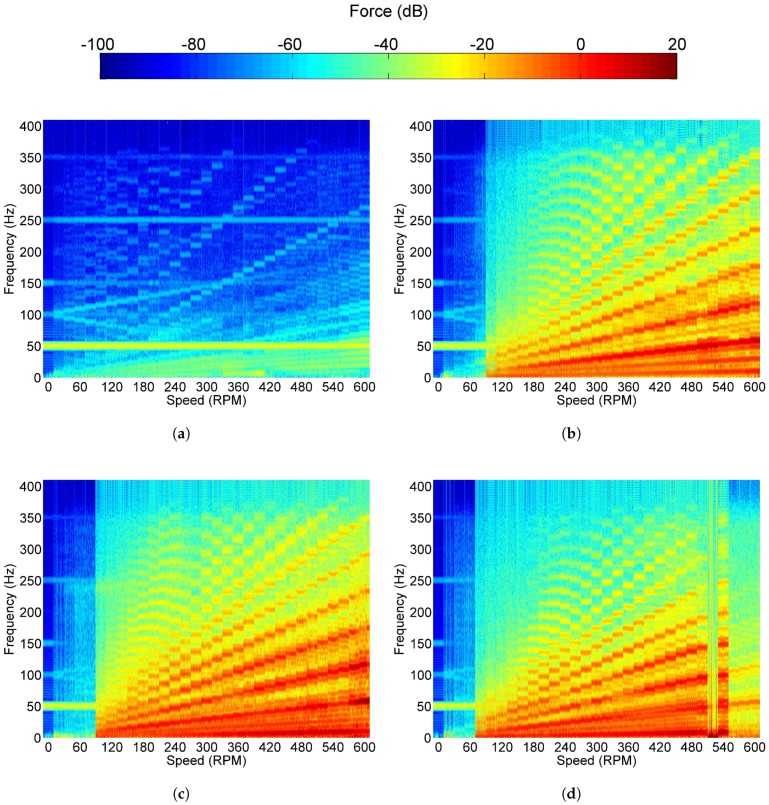
Spectrograms of the PVDF output with: (**a**) of the shaft alone; (**b**) the two-blade propeller; (**c**) the brass two-blade propeller and (**d**) three-blade propeller is attached using the six pad bearing.

The plain shaft spectrogram is shown in Figure 8a. Due to how the spectrogram was generated, bands can clearly be seen which correspond to the 10 second recording for each speed. The features depicted are due to the shaft spinning in the bearing with no load applied. A prominent 50 Hz noise is noticeable across the measurements. Additional components of 150, 250 and 350 Hz which are independent of shaft speed are also seen. The shaft rate and its harmonics can be seen originating from 0 Hz and increase linearly with speed. In addition to this, speed dependent signatures can be seen originating at 100 Hz. The bifurcation is likely due to the electromagnetic noise of the electric motor, as when a nominal shaft rate of 0 RPM is selected, 100 Hz noise is still observed, and noise will vary with selected shaft rate.

The resulting spectrum when the two, two-blade propellers were attached to the end of the shaft are shown in Figure 8b,c. From the Figure, the previously discussed engagement force required for the thrust bearing can clearly be seen in both data sets, which is achieved at 100 RPM. Below this speed, the spectrogram is very similar to that of just the shaft spinning. The 50 Hz electromagnetic noise is present, however, compared to the shaft rate and the PPF, this is considered reasonable. The shaft rate can be seen with its harmonics, however, the largest signature seen is six times the shaft rate which corresponds to the PPF of the system. The two propellers produce similar spectrograms, which is to be expected as the excitation frequencies will be similar, however, there are slight differences concerning their magnitudes.

The final configuration, the three-blade propeller, is shown in Figure 8d. The engagement force required for this configuration is achieved at a slower shaft rate of 80 RPM. The inconsistencies seen in Figure 8d at 520 RPM are a result of delamination of the PVDF sensor (see Figure 7 and associated discussion). The sensors appear to recover at 540 RPM but fail again at higher speeds.

These tests were repeated with different pad configurations. The results obtained where consistent and the most relevant data is shown here.

**Figure 9:**
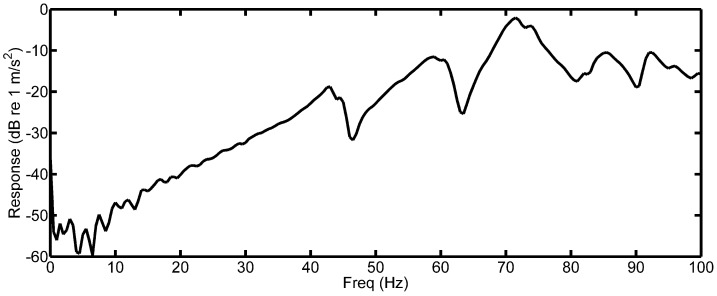
Supporting beam frequency response function.

The response measured during the impact test of the supporting plate is shown in Figure 9. It is clear that the response of the plate increases with excitation frequency. This suggests that higher order BPF and PPF excitation will result in a larger response from the strain gauge when compared to the PVDF measurement.

**Figure 10:**
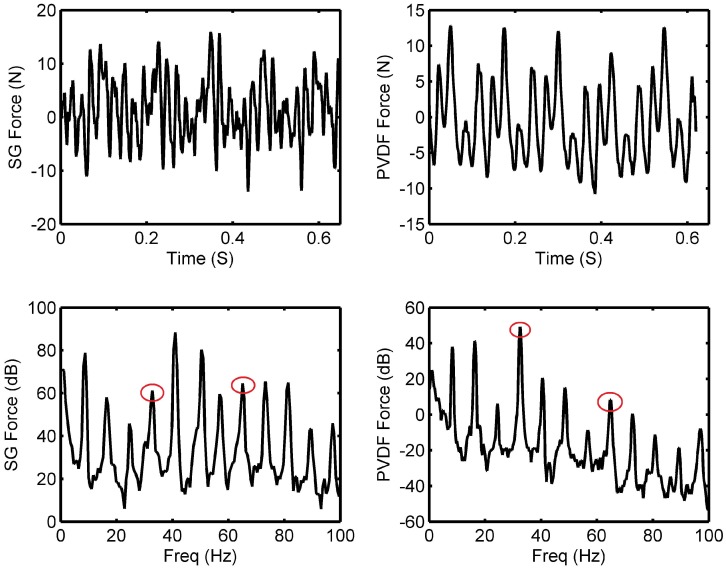
Comparison of the predicated force of the thrust bearing to the localised force measured by the PVDF film for the brass two-blade propeller, four Pad thrust bearings rotating at 500 RPM. Where (top) is the time domain and (bottom) is the frequency domain. The circles indicate the PPF and the modulation frequency.

It was possible to compare the thrust force estimated externally via the strain gauge with that arising from the PVDF sensors using the approach given in the Appendix A, this is shown in Figure 10 where the two-blade brass propeller was spun at 500 RPM with the four pad bearing. It should be noted that the two forces are not equivalent as the PVDF film is measuring the force applied in a localised area of the film as the pad move over it, while the external measurement represents the resultant force of all the pads acting on the thrust bearing. Observing the two signals enables a comparison to investigate whether the same forcing level is observed, however, the PVDF data should include additional information concerning the pressure profile that is formed inside the bearing which would not be seen externally. This can clearly be seen in the frequency domain where the shaft rate (8.33 Hz) and the 4th harmonic of the shaft rate is the largest in amplitude. The force measured from the strain gauge senses the higher frequency fluctuations of the propeller force which is not seen in the PVDF. This is because the PVDF sensors are distributed sensors that sense across their area; this limits the PVDF sensors ability to measure higher order frequency content when embedded in a rigid housing resulting in a low pass filtering effect. The low frequency features can clearly be seen by the PVDF as this is dominated by the pressure of the sliding pads at a much lower frequency. The low frequency fluctuation of the propeller due to the non uniform wake can be seen in both strain gauge and PVDF data.

Comparing the time domain signals, it can be seen that the magnitudes are similar in value. No other conclusions can be made as the ramp-on and ramp-off of the bearing pressure is not observed outside of the bearing. This effect is also seen in our previous paper Reference [7]. In the frequency domain, both the force obtained from the strain gauge and the PVDF show the shaft rate clearly at 8.33 Hz and its harmonics. At the BPF of 16.67 Hz, the PVDF peak is greater than the fundamental shaft frequency but the inverse is true for the strain gauge results. This may be attributed by a small shaft alignment issue which would impact the strain gauge results far greater. The peak at the PPF, which is four times the shaft rate (33.33 Hz), is larger than adjacent peaks for the PVDF data as this signature is heavily dominated by the pads. This is not the case regarding the strain gauge measurements where the 5th harmonic is greater. This is explained by the increase in response of the beam at these frequencies, this is shown in Figure 9. As the blade passing excitation is coupled with the pads, it is expected that the multiple of the two would also be prominent i.e., modulation. This combination of excitation can be seen where the 8th harmonic of the shaft rate (66.67 Hz) is the most prominent when compared to its adjacent peaks. The same experiment was conducted with a five and six pad bearing with the same pad angle. The same trends were seen. Given the large shaft diameter and close supports, it can be shown that the lowest critical speed of the shaft is far greater than the operational speeds used here.

Due to the configuration of the PVDF sensors in the thrust bearing, the true propeller force is carried by the signatures of the apparent periodic pad passing force. This makes separating the force solely due to the propeller loading from the pad passing force difficult. This task is made more difficult by the fact that the BPF and the PPF are the same, making the contribution from each mechanism indistinguishable. Developing a PVDF bearing arrangement that can permit intrinsic propeller axial force measurement is recognised as a future research need.

## 4. Conclusions

Our previous work [7] showed that by embedding thin PVDF piezoelectric films in the base of a model thrust bearing, it is possible to measure the pressure fluctuations. Here, we embedded the same PVDF films inside an operational thrust bearing. By using a test rig, it was straightforward to investigate the effects of different speeds, propellers and pad numbers. The results showed the blade passing frequency (BPF) and the pad passing frequency (PPF) where readily identifiable. Although the contact force inside a fluid filled bearing is shown, due to the configuration of the bearing and the PVDF film placement on the stationary collar, the BPF is superimposed on the PPF. Developing an arrangement and signal processing technique that can permit straightforward BPF and shaft axial vibration measurement is an area of future work.

## Figures and Tables

**Figure A1:**
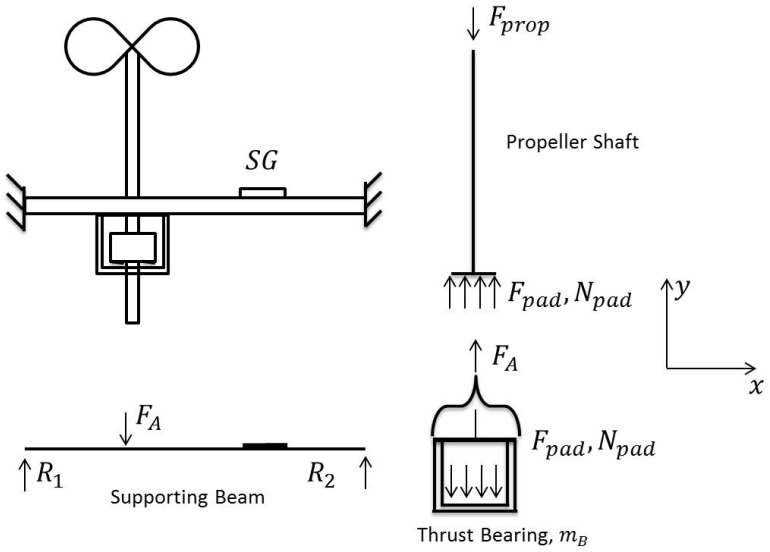
Schematics of the propulsion system (**top left**) and the free body diagrams (FBD) of the propeller shaft (**top right**), thrust bearing (**bottom right**) and the supporting beam structure (**bottom left**).

## Appendix A. Inferring Propeller Force from Strain Gauge Measurement

The zero rotational speed contact force can be determined form the calibrated PVDF sensor (6.8 mV/N). The PVDF signal can be compared to the internal thrust force measurement from the strain gauge data and acceleration signal. To obtain the force from the strain gauge, a calibration factor needs to be determined. This can be achieved analytically and experimentally. To derive the analytical solution, it was assumed that the beam was clamped-clamped as shown in Figure 3. As the diameter of the hole for the shaft is significantly smaller than the plate’s other dimensions, its presence was ignored.

The strain (ε) at the location of the strain gauge is given by Equation (A1) [18].

(A1) $$\varepsilon =\frac{Y}{\rho_{r}}$$

where *Y* is half the beam thickness, and $\rho_{r}$ the radius of curvature due to the beam deflection. $\rho_{r}$ can be calculated from:

(A2) $$\rho_{r}=\frac{EI}{M}$$

where *E* is the Young’s Modulus, *I* is the second moment of area and *M* is the moment about the point of interest. The moment at the location of the strain gauge is given by Equation (A3) [18].

(A3) $$M=\frac{Pab^{2}}{L^{2}}+P\left(L-c-a\right)-\frac{Pa^{2}b}{L^{2}}$$

where P is the force applied at the thrust bearing location, a, b, c and L are the distances shown in Figure 3. Combining Equations (A1), (A2) and (A3) and substituting the dimensions of the rig and the properties of steel results in:

(A4) $$P=18.1e^{6}\varepsilon$$

In a separate experiment, a load cell was attached to the end of the lever arm as shown in Figure 3 and a force applied to generate an axial propeller force at the propeller hub. The force from the load cell and the strain from the strain gauge were recorded using a DigiDAQ system. This system is comprised of eight acquisition units that are connected to a base station via wired Ethernet. The unit provides strain gauge excitation, signal conditioning and analogue to digital conversion.

To evaluate the performance of the PVDF film as a force transducer, the film’s predicted force was compared to that of the force predicted by the strain gauge and accelerometer reading using the model developed from the free body diagrams in Figure A1.

The system is broken up into three sub-systems. The first being the propeller shaft coupled to the thrust washer, where $F_{prop}$ is the force due to the propeller, $F_{pad}$ is the reaction force due to the oil pressure under a single pad in the thrust bearing and $N_{pad}$ is the number of pads. The second subsystem is the thrust bearing which has mass $m_{B}$. The supporting beam is the final sub system, which is assumed mass less.

From the thrust bearing FBD analysis:

(A5) $$\begin{array}{c} F_{pad}=\frac{F_{A}-m_{B}\ddot{y}}{N_{pad}} \end{array}$$

Equation (A5) links the force applied by the thrust bearing pads to the strain gauge and accelerometer measurements. The result of the experiment to determine the strain gauge sensitivity to a applied force at the end of the shaft found it to be 17.8 μN., which is within 2% of the theoretical value. It should be noted that the previously discussed analysis assumes a static load is applied to the beam, resulting in a static deflection. It is expected that a dynamic analysis would result in a frequency dependent strain gauge sensitivity due to the beam’s response, however, a static approximation was deemed sufficient.

## References

[1] Dylejko P.G., Kessissoglou N.J., Tso Y., Norwood C.J. (2007). Optimisation of a resonance changer to minimise the vibration transmission in marine vessels. J. Sound Vib..

[2] Sawicki J.T., Rao T. (2004). A nonlinear model for prediction of dynamic coefficients in a hydrodynamic journal bearing. Int. J. Rotating Mach..

[3] Tiwari R., Lees A., Friswell M. (2004). Identification of dynamic bearing parameters: A review. Shock Vibr. Digest.

[4] Zhang S., Zhang Q. Coupled torsional and axial nonlinear vibration model of the crankshaft with a propeller. Proceedings of the 2008 Asia Simulation Conference–7th International Conference on System Simulation and Scientific Computing.

[5] Pan J., Farag N., Lin T., Juniper R. Propeller induced structural vibration through the thrust bearing. Proceedings of the Annual Conference of the Australian Acoustical Society.

[6] Parkins D.W., Horner D. (1993). Tilting-Pad Journal Bearings–Measured and Predicted Stiffness Coefficients. Tribol. Trans..

[7] Youssef A., Matthews D., Guzzomi A., Pan J. (2017). Measurement of pressure fluctuations inside a model thrust bearing using PVDF sensors. Sensors.

[8] Lee I., Sung H. (1999). Development of an array of pressure sensors with PVDF film. Exp. Fluids.

[9] Talbot J.P. (2002). On the Performance of Base-Isolated Buildings: A Generic Model. Ph.D. Thesis.

[10] Kim J., Park Y., Choi I., Kang D. (2002). Development of Smart Elastomeric Bearing Equipped with PVDF Polymer Film for Monitoring Vertical Load Through the Support. VDI Ber..

[11] Shirinov A., Schomburg W. (2008). Pressure sensor from a PVDF film. Sens. Actuators A.

[12] Grinspan A.S., Gnanamoorthy R. (2010). Impact force of low velocity liquid droplets measured using piezoelectric PVDF film. Colloids Surf. A.

[13] Mahale B.P., Bodas D., Gangal S. Development of PVDF based pressure sensor for low pressure application. Proceedings of the 6th IEEE International Conference on Nano/Micro Engineered and Molecular Systems.

[14] Nash B.T. (2012). Pressure Mapping Using PVDF Film. UNSW Canberra ADFA J. Undergrad. Eng. Res..

[15] Zhang J., Wang Y. On the desien of intelligent insoles using PVDF film. Proceedings of the Symposium on Piezoelectricity, Acoustic Waves, and Device Applications (SPAWDA).

[16] Measurement Specialties (2008). Piezo Film Sensors Technical Manual.

[17] Stachowiak G., Batchelor A.W. (2013). Engineering Tribology.

[18] Shigley J.E. (2011). Shigley’s Mechanical Engineering Design.

